# The Personal Emergency Response System as a Technology Innovation in Primary Health Care Services: An Integrative Review

**DOI:** 10.2196/jmir.5727

**Published:** 2016-07-14

**Authors:** Randi Stokke

**Affiliations:** ^1^Centre for Care ResearchNorwegian University of Science and Technology (NTNU)GjøvikNorway; ^2^The Centre for Innovation in ServicesLillehammer University CollegeLillehammerNorway

**Keywords:** home care services, caring practice, personal emergency alarm system, PERS, safety alarm, social alarm, telecare, review

## Abstract

**Background:**

Most western countries are experiencing greater pressure on community care services due to increased life expectancy and changes in policy toward prioritizing independent living. This has led to a demand for change and innovation in caring practices with an expected increased use of technology. Despite numerous attempts, it has proven surprisingly difficult to implement and adopt technological innovations. The main established technological innovation in home care services for older people is the personal emergency response system (PERS), which is widely adopted and used throughout most western countries aiming to support “aging safely in place.”

**Objective:**

This integrative review examines how research literature describes use of the PERS focusing on the users’ perspective, thus exploring how different actors experience the technology in use and how it affects the complex interactions between multiple actors in caring practices.

**Methods:**

The review presents an overview of the body of research on this well-established telecare solution, indicating what is important for different actors in regard to accepting and using this technology in community care services. An integrative review, recognized by a systematic search in major databases followed by a review process, was conducted.

**Results:**

The search resulted in 33 included studies describing different actors’ experiences with the PERS in use. The overall focus was on the end users’ experiences and the consequences of having and using the alarm, and how the technology changes caring practices and interactions between the actors.

**Conclusions:**

The PERS contributes to safety and independent living for users of the alarm, but there are also unforeseen consequences and possible improvements in the device and the integrated service. This rather simple and well-established telecare technology in use interacts with the actors involved, creating changes in daily living and even affecting their identities. This review argues for an approach to telecare in which the complexity of practice is accounted for and shows how the plug-and-play expectations producers tend to generate is a simplification of the reality. This calls for a recognition that place and actors matter, as does a sensitivity to technology as an integrated part of complex caring practices.

## Introduction

### Background

Western societies have an ageing population due to increased life expectancy and large cohorts in the postwar years, presenting growing challenges to long-term care services [1,2]. Independent living for older people is a policy priority in western countries [3], and this includes active ageing and maintenance of quality of life [4,5].

Meeting such demands through technology innovation is one suggested solution. Technological innovations in community care services are highly regarded, even though integration and adoption has proven difficult and many projects never pass the pilot stage [6,7]. Studying how different actors perceive and experience existing technologies in use is one way of providing a richer and more nuanced view of what promotes or inhibits adoption of new technology innovations [8]. This review seeks to do so by exploring research on the personal emergency response system (PERS). The PERS is a widespread, integrated, and accepted technology innovation in care practices. Through focusing on the users` perspectives, this review explores how different actors experience the technology in use and how it affects the complex interactions between multiple actors in caring practices.

### The Personal Emergency Response System

The PERS has proved sustainable over time. Since its launch in the late 1970s, it is widely used and spread throughout most western societies [9]. For example, there are approximately 1.4 million users in the United Kingdom [10] and 74,000 users in Norway [11]. Even after many years of use, no review article summarizing use of the PERS research has been identified. In fact, the research literature on the subject is rather sparse [12].

The PERS is a technological device and an integrated service, embodying three generations of alarm devices as a result of technical development, although some characteristics remain unchanged. The first generation alarm device had a unit placed centrally in the home, with a switch or a pull cord to use in emergency. The second generation has in addition a pendant, a necklace/wristband with a button that the user can press in case of emergency. This allows open communication between the user and a responder through the main unit, enabling the responders to effect a proper response. The range of the pendant is normally inside the home and partly outside. The third and newest generation of the PERS has the potential to incorporate a range of devices (eg, automatic fall alarms, fire alarms, and blood pressure devices [10,13]), providing remote care [14].

It is mainly the second and third generation versions that are in use today, although implementation of additional alarms, devices, and services has proven difficult and is done on a much smaller scale than expected with a slowly growing market. Thus, the PERS might be described as a foundation for further safety and monitoring telecare.

The organization of the PERS as a service varies from private arrangements, where the alarm goes to a nominated contact, to small or large public or private call centers answering and effecting proper responses [1,10]. The PERS as a service system is complex, dealing with a variety of contexts and services [15], and its organization and use are part of integrated caring practices with multiple actors. The different actors are, among others, the end user and their relatives and neighbors, home care nurses, and telecare facilitators. They are all users of the PERS but have different experiences, roles, meanings, and relationships with each other and the technology. The end user of the PERS with the alarm in his/her home is usually an older person [11,16].

### Use of Technology in Caring Practices

Policy makers and advocates of such alarm systems often describe telecare technologies such as the PERS as ‘‘plug-n-play’’ solutions with placement of devices at home providing help in an effective way, enhancing quality of life, and reducing costs for the care service [15]. However, the use of telecare tends to be more complex than such promises suggest [17]. There are many indications that moving away from the rather naïve technological determinism, where telecare technologies are simply viewed as plug-and-play devices, and instead acknowledging the complexity in technology practices would provide a more accurate view of practice.

### Theorizing Use of Technology in Caring Practices

When studying practice, Nicolini [18] argues that the purpose of social science is to open up for a rich and nuanced understanding of practice [18]. He argues that there is no such thing as a unified practice theory and suggests using what he calls a toolkit approach by mobilizing different aspects of similar theories when exploring practice. This enables enriched understanding of what is going on. He suggests what he calls a “theory-method package,” which when utilized in this review involves zooming in on the practice of the PERS in use as displayed in the included articles, and zooming out following trails of connections. By zooming out, it is possible to draw on the local practice of the PERS in use to acquire a wider picture of technology in use in caring practices. Scholars such as Nelly Oudshoorn [19-21], Jeanette Pols [15,22,23], and Davide Nicolini [18,24,25] have studied different kinds of telecare that will provide tools for zooming out, exploring and theorizing technology in use in caring practices. Both Nicolini and Oudshoorn are inspired by science and technology studies (STS) and the fields of human geographies aiming to bridge these approaches by focusing on how place is important when shaping user and technology relations [18,20,24]. Technology, actors, society, and place must be thought of together since they are coconstructed, and technology is by definition technology in use.

Three main questions exploring how technology in use is integrated in caring practices arise from what research tells us about the users’ experiences with the PERS:

1. What has research focused on when studying use of the PERS over time?

2. How do actors in home care practices experience, integrate, and relate to the PERS in everyday life?

3. How does this established technology in use influence and affect caring practices?

## Methods

An integrative review was conducted, characterized by explicit, rigorous, and transparent methodology using a systematic search, but allowing the inclusion of research with diverse methodologies and a broader range of studies [26]. Ethical approval was not required as this was secondary research. Textbox 1 presents inclusion criteria applied to articles before the searches.

**Inclusion criteria for articles to be included in the review.**

Inclusion criteria:

- Articles dealing with older people’s attitudes, experiences, interactions, feelings, use and nonuse, consequences, and effects of use of a personal emergency response system (PERS) in home care
- Peer-reviewed articles from academic journals describing and focusing on different aspects of the PERS in use rather than articles with a main focus on further technology innovation
- Articles written in the English language, no limitation in publication period, and no methodological restrictions

**Figure 1:**
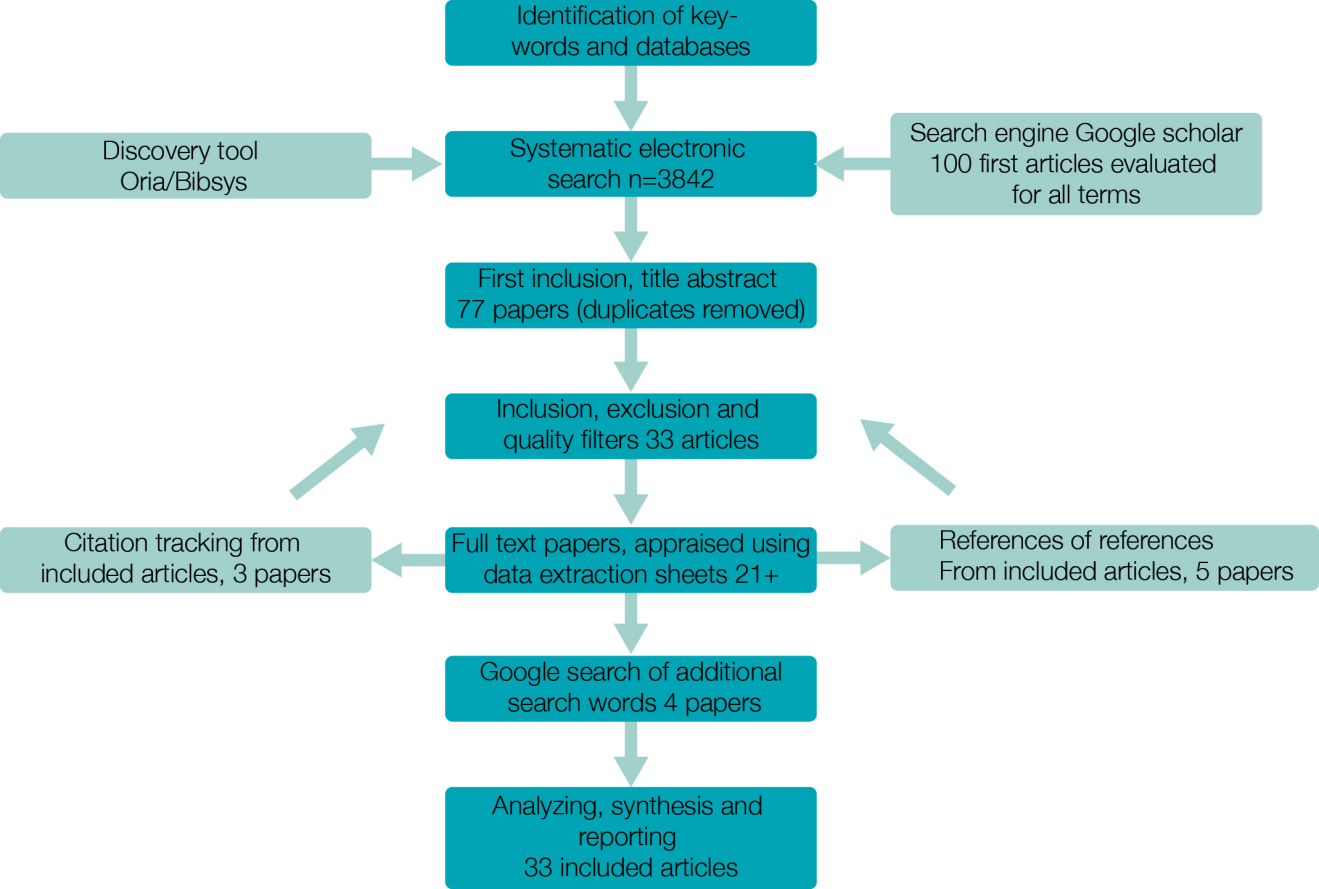
Overview of the article searching process.

A systematic search of relevant terms was conducted in relevant databases and search engines. The search strategies and results are presented in Figure 1. All articles were reviewed according to the inclusion criteria. A thorough description of the steps describing the research strategy process is described in Multimedia Appendix 1. A data extraction sheet was a useful tool for quality in assessing the articles.

A descriptive, integrative, thematic analysis as described by Whittemore and Knafl [26] was used to analyze the articles. This required ordering, coding, categorizing, and summarizing data [26]. Table 1 presents a comparative and systematic organization of the included studies [9,16,22-52].

The next steps were exploring and displaying the extracted data around different variables and subgroups looking for patterns, themes, and relationships, and then drawing a map of the essential identified themes. This was followed by abstracting and grouping themes into categories, aiming to subsume the particulars into more general findings. To ensure quality, the included articles were checked to verify for accuracy and conformability. Uncertainties throughout the process were discussed with a group of supervisors. Methodological considerations are described in Multimedia Appendix 2.

**Table 1:** An overview of the included articles in this integrative review.

Article	Country	Methods	Main findings	Term used
Boström et al 2011 [42]				
	Sweden	Focus group interviews with PERS^a^ users	The participants’ opinions and feelings with the PERS related to five themes: safety, anxiety, satisfaction, information, and older persons as active innovators.	PERS
De San Miguel and Lewin 2008 [43]				
	Australia	Mail survey to 2610 PERS users	Clients reported impacts on emergency response, living independently, sense of security and anxiety, and when and where they wear their alarm.	Personal alarms
Fallis et al 2007 [33]				
	Canada	Mixed-method design, survey, and qualitative feedback	Need for improvement. The PERS gave sense of security, comfort, and reassurance, with high satisfaction with service during an emergency.	PERS
Farquhar et al 1992 [47]				
	Australia	Assessment intervention with 125 persons	Respondents described high satisfaction with the alarm. Total of 38% gave up the alarm; 62% never used the alarm, but 84% felt they required it.	Personal emergency alarms
Fisk 1995 [38]				
	United Kingdom and Canada	Qualitative interviews with 38 users from Oldham and Ottawa	A majority experienced a feeling of security; 40-50% had used system in emergency. The alarm was not always used in emergencies.	Personal response services
Fleming and Brayne 2008 [48]				
	United Kingdom	1-year follow-up of 110 patients	Total of 54% of reported falls happened when person was alone; 80% did not use alarm to summon help. Users described different barriers.	Call alarm system
Heinbüchner et al 2010 [9]				
	Germany	333 PERS users approached; response rate 19.6%	Respondents were satisfied with their device, although 24% never wore the pendant. The PERS was not activated by 83% of the persons who fell.	PERS
Hyer and Rudick 1994 [44]				
	United States	Telephone survey of 117 patients monitored; maximum 1 year	One-third of PERS users requested emergency assistance (60 calls); significant cost savings; high patient satisfaction.	PERS
Johnston et al 2010 [35]				
	Australia	31 semistructured interviews	Identified four subgroups: 1) used alarm effectively, 2) had alarm, but not used effectively, 3) no alarm, but were receptive, 4) no alarm and would not use it.	Personal alarms
Johnston et al 2010 [37]				
	Australia	1-month retrospective audit of 1700 cases (alarms)	Difficult to separate false alarm from emergencies. Personal alarm might be helpful for people living alone, when alarm is accepted, understood, and used effectively.	Personal alarms
Lee et al 2007 [45]				
	Canada	RCT^b^; recruited after admitted to ED^c^ after fall	There was no difference in mean change in anxiety between the groups. Alarm user had decreased fear of falling.	PERS
Levine and Tideiksaar 1995 [49]				
	United States	Structured interviews; 106 participants	Total of 45% of respondents were fully compliant; identified factors that increased compliance.	PERS



Mann et al 2005 [50]				
	United States	Surveyed 606 people; users and nonusers of PERS	Total of 92.7% were satisfied with their PERS; 84.3% rated their PERS as very important. The major reason for potential use was falling and feeling ill.	PERS
McWhirter 1987 [51]				
	United Kingdom	Quantitative client register questionnaire; 667 females, 194 males	Main reason for referral: problems with mobility (45.6%) and falls (43.4%); 40% of all calls were false alarms.	A dispersed alarm system
Melkas 2003 [55]				
	Finland and Sweden	40 interviews with service personnel	The study is mainly about information systems around use of the PERS; bottlenecks are identified.	Safety telephone services
Melkas 2010 [56]				
	Finland	Human impact assessment methodologies	Total of 8 care workers at 8 workplaces. Changes, problems, and strengths related to information environment; improving information environment.	Safety telephone services
Nyman and Victor 2014 [41]				
	United Kingdom	A secondary analysis from an English study of ageing	Investigated self-reported users of personal call alarms among 3091 adults aged 65+. From a large sample of those aged 65+, use of call alarm was rare.	Personal call alarms
Olsson et al 2012 [39]				
	Sweden	Interview with 14 spouses of persons with dementia	Total of 4 spouses had safety alarm; used for different purposes (eg, if person with dementia had fallen or suddenly fell ill and they needed help).	Safety alarm
Pekkarinen and Melkas 2010 [16]				
	Finland	Mixed methods; qualitative interviews; survey with users and personnel	Describing different “potholes” in the technology, service, process, organization, marketing, and ethics and how these can be dealt with.	Safety alarm systems
Porter 2003 [27]				
	United States	56 qualitative interviews with 8 widows	Experiences of having the PERS. The findings were a basis for considering the potential influences of having a PERS on older people’s well-being.	PERS
Porter 2008 [28]				
	United States	Phenomenology; semistructured interviews with 14 women	How the PERS influenced what older people would do if an intruder got in their house.	PERS
Porter and Lasiter 2012 [29]				
	United States	Phenomenology; part of a larger RHQ^d^project; 95 interviews with 25 women	Life-world being influenced by a peer’s situation regarding adopting or using a PERS for reaching help quickly.	PERS
Porter 2005 [30]				
	United States	Phenomenology; interviews with 7 frail women during 3 years	The women’s experiences of PERS; a description of temporizing about the PERS button—deciding when to wear it and whether to use it.	PERS
Porter 2002 [31]				
	United States	Phenomenology; part of a longitudinal study; 71 interviews of 11 widows	Experiences of not having the PERS; exploring reasons and barriers for PERS use.	PERS



Porter et al 2013 [32]				
	United States	Phenomenology; 99 interviews with 23 women	PERS subscribers’ and nonsubscribers’ intentions and context differ relative to reaching help quickly (RHQ).	PERS
Premik et al 1997 [53]				
	Slovenia	Quantitative data from the PERS	Total of 18,500 alarm calls in 4 years; 2.1% health related. The alarm could be a basic communication device for older people.	Community social alarm system
Raappana et al 2007 [57]				
	Finland	Human impact assessment methodology; 8 workplaces, 78 care workers	Safety alarms might be useful both for administration and actual care work.	Safety alarm system
Roush and Teasdale 2011 [34]				
	United States and Canada	Survey; 267 older persons	PERS users utilized emergency departments twice as often as those without. Strong relation between access to a PERS, sense of security, and higher levels of well-being.	PERS
Roush et al 1995 [40]				
	United States and Canada	Hospital utilization rates; 106 patients; 1-year follow-ups	PERS users had a significant decrease in per-person hospital admissions and inpatient days. No significant differences in ED visits.	PERS
Sjölinder et al 2014 [52]				
	Sweden	Mixed-methods survey, interviews, and focus groups	The municipalities’ knowledge about the new technology was deficient. Focuses on possibilities for using alarms outside.	Social alarm system
Tinker 1993 [36]				
	United Kingdom	Literature summary from two major reports	Summary findings from two reports.	Dispersed alarms
Vincent et al 2006 [49]				
	Canada	Quantitative quasi-experimental design; 975 calls for 38 clients over 6-month period	Positive effect on caregiver burden. Number of home visits by care workers decreased. No improvement in quality of life.	Tele-surveillance
Youssef et al 2000 [41]				
	United Kingdom	Quantitative study; recorded calls to a control center for 6 months	Total of 542 alarms excluding false alarms. Caregiver solved most problems. GP^e^ was called on 38 occasions, ambulance called on 91 occasions, 44 transported to ED, and 29 admitted.	Community alarm

^a^PERS: personal emergency response system.

^b^RCT: randomized controlled trial.

^c^ED: emergency department.

^d^RHQ: reach help quickly.

^e^GP: general practitioner.

## Results

### Overview

A total of 33 peer-reviewed articles were included, all published between 1987 and 2014 [9,16,22-52]. The articles differ in purpose and study design and represent a wide range of methodology and research traditions. There has been a methodological development in the field. Simplified, descriptive, quantitative evaluation studies of predefined effects dominated the early studies. From the year 2000, we see both qualitative and quantitative studies increasingly displaying a perception of the complexity of the service and society, for example, in-depth views of frail old women’s personal experiences with the PERS through the phenomenological studies of Porter et al [27-32].

Table 1 shows that out of 33 studies, 20 (61%) focused directly on the end user, and were related to different aspects of having a PERS. A total of 5 studies (15%) used registered data from the alarm centers regarding use, malfunction, etc. A total of 2 studies (6%) looked mainly at how use of the alarm affects emergency admission, response time, and economy. The remaining studies focused on the service system, the service provider’s experience, and the service organization.

As shown in Table 1, different terms are used for the alarm system. The *personal emergency response system (PERS)* is the term most commonly used in articles from the United States, Australia, Germany, the United Kingdom, and Sweden. Other common terms mainly used in the United Kingdom, Scandinavia, and Australia are variations of *personal*, *safety*, *social*, and *community alarms*.

The following section will follow Nicolini's [18] suggestions of “zooming in” on practice, what people do or say, patterns of relationships, and what mechanisms achieve durability in time. This is done by focusing on the included studies' descriptions of the end users’ experiences with the PERS, followed by other actors’ experiences. Thereafter, I will describe how the included studies describe the interaction between the human actors and the technology, as well as the different actors’ wishes for future telecare.

### The End Users’ Experiences With the Personal Emergency Response System

Summing up the demographic data from the included studies, the typical PERS end user is an old, fragile woman, living alone, over 80 years of age with physical problems and in need of assistance. The articles state that the end users find the alarm easy to use. Only 2 out of 33 studies (6%) describe demands for training and information and suggest that short learning sessions are preferable to one initial, long session [16,33].

Even though most studies indicate the usefulness of the alarm for fragile elderly people, the alarm does not seem suitable for everybody. Roush and Teasdale [34] found that it is difficult to establish who would utilize a PERS. It is a useful way of getting help faster when the alarm is accepted, understood, and used [35]. A significant proportion of the elderly are less likely to utilize the PERS when in need, especially confused persons [13,36]. Inability to press the button, forgetting to wear it, failing to remember that one is wearing it, or being unable to let helpers inside are reasons given for end users not using the alarm [31,37].

The most stated reason for getting a PERS was the possibility of getting help fast in an emergency [27,30,32,33,35,38-40]. Living in isolation, poor mobility, experiences with hospital stays after a “long lie” following a fall, and concern for personal safety were all catalysts for acquiring the alarm [29,35,37,41].

The articles found that many end users were satisfied with the PERS overall, since it enabled them to summon help if necessary, and that staff were patient despite false or repeated alarms [9,31,33,34,38,42-44]. The studies reported success stories involving activation of the alarm [33]. The ability to get help faster provides a sense of security. Roush et al [40] found that PERS users experienced higher levels of well-being. Although a randomized controlled trial (RCT) assessing the impact of a PERS on anxiety found there was a slightly decreased fear of falling, there was, however, no reduction in anxiety [45]. Another study found that regardless of positive experiences, there was no significant improvement in quality of life [46].

Almost all included studies discussed reasons for activation of the alarm. They demonstrated quite different results regarding the frequency of activation due to emergencies. In one study, only 2.1% of alarm activations were due to emergencies whereas in another, the figure was 67%. Falling was the most common reason for emergency calls, and many users had fallen more than once. Other medical emergencies were also common [33,36,38,43,44,46-51]. End users with a positive experience from a previous emergency were more compliant and satisfied, and men were more likely than women to use the alarm more frequently [33,41,49].

Despite satisfaction with the service, the studies found challenging experiences for the alarm users as well. One study describes fear and insecurity regarding whether the PERS would function when needed, especially at nighttime [42]. The PERS was found to increase ability to live independently [43], and having the alarm was of importance in maintaining end users’ lifestyles. Some stated that the PERS helped them to keep their social networks intact [27] and to resume activities they had enjoyed previously [38]. Other studies reported a negative effect on the end user’s social life due to uncertainty about the pendant range [37]. To feel safe, the solution was to stay indoors [16,42,52].

Nyman and Victor [41] found that there was not necessarily a correlation between the perception of being satisfied/thinking it is important and actually wearing or using the alarm. They found that the PERS was highly accepted but rarely used. The included studies reported rather different results regarding whether the respondents wore the alarm pendant. End users who considered the PERS important for them wore the pendant significantly more [9,43,47,50]. A total of 2 studies out of 33 (6%) found that about 25% of the respondents never wore the pendant [9,34].

According to the articles, the end users had many reasons for not wearing the pendant: “forgot to put it on,” “worry it will get damaged,” “do not think they need it at the time,” “not satisfied with the PERS,” and “uncomfortable to wear” [27,30,45]. Porter [30] found that all women interviewed who wore the pendant did so unwillingly. The PERS made it possible to live alone, but also made life more complicated due to choices as to when to wear and activate the alarm, and fear of triggering it by accident.

It is clear that the alarms are also used for purposes that were not foreseen or intended [33,38,43,45,53,54]. Porter [28] found that some end users would use their PERS if an intruder came, believing that the loud voice when connecting would scare off a burglar. The number of false alarms varied considerably between the studies, and so did what counted as a false alarm.

Several studies found that some users would not activate the PERS even in emergencies [9,30,34,48]. Reasons given were as follows: “wanted to manage on their own,” “forgot,“ “call neighbor,” “see if it passes,” “don’t want to be dragged off to hospital,” “afraid to bother,” “called 911,” “unsure whether serious enough emergency,” “don’t want strangers in the house,” and “unsure of helpers’ qualifications” [16,29,32,38,43]. Many respondents never felt in need, and therefore never activated their alarm [47]. There was little focus in the articles on how end users assessed the appearance of the PERS pendant, although there were comments on ”stigmatizing“ appearance in 5 out of 33 studies (15%) [16,30,38,42,54].

Economic issues were mentioned, mainly in studies from the United States and Canada. Users often paid a fee for having the PERS. Both users and responders raised concerns about the costs and felt it to be too expensive [27,33,49,50]. A total of 2 out of 33 studies (6%) described how some users would like to have the PERS but could not afford it [33,35].

### How Other Actors Experience the Personal Emergency Response System

The included studies focused very little on how relatives experienced the PERS. Some studies mentioned that having the alarm gave families peace of mind and reduced their burden [27,30,33,35,38,40,43]. Studies in which private persons were first responders reported that most were happy to remain so [33,47] with the exception of the study of Sjölinder and Avatare Nöu [52].

The studies described different service organizations, from directly distributing the alarm call to a nominated contact, to larger or smaller private or public response centers. Control center operators’ tasks varied according to the service offered. Some response centers were staffed with health care workers [33,47,53,55]. The staff at different call centers had different experiences and attitudes toward the PERS. One large study in Finland found several bottlenecks in the service [55]. Care workers described how the alarm sometimes caused harm and extra work due to accidents, technical failures, and difficulties separating false alarms from emergencies [37]. Experiencing bureaucratic and organizational challenges, they found the alarm to be stressful, costly, and difficult. Others had positive experiences with increased work motivation and better workload planning with reduced visits to end users. The end users gained more privacy and received help only when needed, making night shifts easier [16,56].

### How the Personal Emergency Response System Affects the Interaction Between the Actors

In the included studies, there was little focus on the interactions between the different actors involved in the PERS. Pekkarinen and Melkas [16] found in their study that the holistic situation of end users was not always understood and well-managed by the service providers [16,56]. Some end users reported a less than satisfactory response from the monitoring center and slow response time [16]. The operators were sometimes impolite or the end users’ needs were underestimated [49]. Misunderstandings caused by dialects or unclear speech, insufficient follow-up after hospital discharge, and fear of being a burden were problems described [37]. A total of 2 out of 33 studies (6%) found that users expressed fear of causing false alarms by setting the alarm off accidentally, resulting in strangers’ voices in their homes [30,31].

Studies described how respondents became motivated to request an alarm in different ways. Health care workers and family were the main source for suggesting a PERS. Respondents became more motivated to use a PERS if health care personnel rather than family suggested this [35,45].

Some articles reported that having the PERS reduced end users’ contact with family [43], leaving them feeling lonely, having only the alarm [56]. Both care workers and end users described fears that technology would replace personal service and the support of friends, family, etc. [16,51]. The PERS allowed users to get help when needed, but there was little description of what “when needed” implied. Some studies described the PERS merely as a medical emergency system [30,35,40,43,49,51,54]. Others indicated that PERS is a service that includes guidance in health and medication questions and social calls in addition to being an emergency system [53,54,57].

As reflected in the name, the PERS is a technical device integrated in a service system. Many of the included articles touched on technological problems, even though this is a well-established technology. There is no connection between the technical failure reported and the age of the studies. In addition to limited and confusing alarm range, reported problems included insufficient speaker capability, battery failure, varying needs for button sensitivity, and nonreplaceable parts of the device [16,33,42,47,52,54,55].

### Wishes for the Future

Some studies described wishes for improvement of the PERS. End users wanted longer pendant range, smaller pendants, and for the PERS to be waterproof, personalized, include global positioning system (GPS) and relevant alarms, automatic connection to the nearest health personnel, and automatic dispatching sound when in need [16,42]. They also suggested how service could be improved by responders identifying themselves and speaking slowly and loudly, and that written materials in large print should be provided [33].

## Discussion

### Principal Findings

The key objectives of this integrative review were to explore existing research on the PERS and to seek insight into how actors experience this technology in use in home care services, thus providing a richer and more nuanced view of how actors interact with technologies in caring practices. By following the *theory-method package*, as described by Nicolini [18], the “zooming in” on the practice of the PERS in use as displayed in the Results section will be followed by “zooming out” in this Discussion section, following trails of connections between the PERS in use and other telecare practices. The focus for further discussion is how terms and place matter, and how different actors interact and create changes in roles, use, interactions, and practices. In this way, we can acquire a wider picture of technology in use in caring practices. This provides us with insights of what makes the practice of the PERS so durable over time and contributes to an understanding of what we can draw from this to other caring practices with telecare in use.

### How Words Create Reality

The many different terms used for this technology may reflect different conceptions of the purpose of the technology. The term *personal emergency response system* indicates that the purpose is to respond to an emergency. The term *safety* or *social alarm* indicates that the alarm might include help with social issues and is there for the end user’s safety. Technologies are scripted, like a play or a film. This means that the designer and producer have context and users in mind when developing technology [58]. Oudshoorn [20] argues that a large part of the practice involving different kinds of telecare involves filling the gaps between the scripts and the technology practice. This diversity in terms, aim, and purpose seems to result in uncertainty among the end users about what is a legitimate use of the alarm as described in several of the included articles. Thus, this illustrates differences in the ways the script of the technology is presented and lived.

### The Privacy of the Home

Oudshoorn [20] argues that implementing telecare in someone’s home creates changes. The home is no longer the same private sphere when connected to health care centers. She describes this as a medicalization of the home. The care personnel only come when the end user for some reason activates the alarm. Even so, the results show how end users’ fears of activating the alarm by accident with the subsequent arrival of “strangers” in the house increases their anxiety. Further development of the passive alarm in connection with the third generation of the PERS challenges the definition of a private home even more, turning the home into a place for monitoring health and daily living. Milligan and Wiles [59] describe this as technologies creating “cracks in the door,” allowing care personal to monitor and enter the home without physical presence [20,59]. On the other hand, this review shows how health care workers relate that they no longer have to disturb the patient’s privacy at night, knowing that he/she will use the alarm if in need. Some end users describe how knowing the connection is there makes them feel safe so that they dare to be more active. Hence, changing the home from a private place to an arena for telecare in caring practices will change the home in complex and contradictory ways.

### Changing Roles in Caring Practices

Telecare promises an opportunity to get help when needed in one’s home. Previous studies showed little reference to the shift this brings about in the redistribution of responsibility among the actors involved, with the delegation of major responsibility to care workers and the end user [21,24]. Use of telecare redefines the patient role from a passive recipient to actively participating in health care and safety monitoring, and demands that patients become competent users of the technology [20]. This demand for active participation largely delegates the responsibility for their own safety to end users. The results of this review found that the PERS, even though it is considered easy to use, is not for everybody. The PERS gives the end user an active role in their care. He/she has to press the pendant to reach help. The results describe how end users do not always remember or manage to activate the alarm. The PERS is therefore not suited for end users who are unable to activate the alarm when in need, for instance, mentally confused people.

This responsibility is further extended in devices developed for the third generation of the PERS, where patients are expected to some extent to manage and monitor their own health. This is a double-edged sword: patients on the one hand gain knowledge and ownership of their own health, but often face demanding requirements to master the technology within the strict frames decided by the system and the technology. This requires that the end user develop skills in using the technology in possibly stressful situations since the caregiver is not present in the home.

These changes in roles, responsibilities, and work were largely disregarded in the studies included in this review. The findings paid little attention to the changes in the role of the care personnel. However, this review reveals a diversity and complexity in health care workers’ experiences with the PERS, varying from finding that the technology enables them to give the users more privacy and freedom, to complicating the organization of the work and causing stress and failure in the caring practice.

### Resistance and Nonuse of Telecare

Promoters of technology innovations tend to describe resistance and nonuse of technology due to lack of technological skills and access among older people as a generational issue [20,60]. Akrich and Latour found that instead of complaining about the technology, actors tend to adjust their practices or resist using the technology [20,58,61].

Despite the main finding of the studies included in this review of users showing great satisfaction with the PERS, the results described how many users acquire the alarm, but hardly ever wear or activate the alarm pendant. As previously described, it seems clear that some nonuse described in the results was related to lack of ability to utilize the alarm, but that is not the whole story. Those not using the alarm often had relevant reasons for doing so. In addition to the previously mentioned reasons, many end users described how they did not feel they needed the PERS, found other solutions for being safe, did not want to bother or be bothered, or found the PERS stigmatizing. It seems that nonuse is more complex than the users’ lack of skills and access, though that is also important. This review shows that resistance and nonuse are due to factors such as the change in caring practices and the way users experience the technology as changing their lives and homes. The results also indicate how end users experience challenges related to use of the PERS, including technological failure, fear of the alarm not working, and limited alarm pendant range.

Pols [15] affirmed that different user groups of telecare tend to be defined by similarities within the group, but she stresses that there are huge differences and heterogeneities within different groups. The studies included in this review described the end users of the alarm as fragile, high-dependence older people, often with an extensive medical history and often living alone; this indicates a need for the PERS. None of the studies discussed diversity within this group of end users, although some described how respondents talk about resistance to being considered “one of those.” It seems that having the PERS defines the end users as part of the group of frail, old, dependent people, and this causes resistance among some.

### How Telecare Creates New Interactions and Practices

Telecare implies a different kind of care with complex interactions between multiple actors and a wide variety of technology and changed roles, thus redefining how actors live, work, and even identify their lives [20]. Results show that having the PERS affected the users in different ways socially. Some became more active because they felt safe having the alarm; others, however, felt restricted by the pendant range and therefore stayed inside their homes. Some actors feared that the alarm might replace human contact.

This review reveals a huge diversity in the experiences and roles of the actors involved, presenting a variety of experiences, both negative and positive, from end users, care personnel, and other actors. Therefore, we cannot really talk about one type of practice related to the PERS, but rather a variety of practices as a result of the interactions between the technology and the actors involved, and how the service is organized and carried out.

The history of integrating telecare in community care shows that the technology in use tends to work in unforeseen and different ways than intended [20]. The results of this review show how the end users activated the alarm for a number of reasons, and found new and other functions for the alarm than the one intended (eg, older women planning to use the PERS to scare away unwanted intruders). Users also had wishes for future functions that would increase the value of having the PERS, for example, increased pendant range, integrated GPS, and smaller pendants, to mention a few.

### What the Technology Does—and What We Think it Does

Pols [15] found in her study of telecare in the Netherlands that use of telecare did not solve existing practice problems but was instrumental in creating new practices with different challenges and problems, and thereby changed the actors’ lives.

The PERS is introduced as technology that makes users safe in their homes and enables them to reach help when needed. This review shows how end users in the included studies expressed satisfaction with the technology, and experienced well-being and a sense of security. However, having the PERS did not reduce anxiety or improve their quality of life. Some of the studies included in this review found that having the PERS did reduce hospital days and medical complications due to long lies after falls. Moreover, the material described success stories involving activating the alarm. To some extent, the alarm thereby fulfilled its promise of increasing safety at home. However, what the results show is that the picture is much more complicated than the PERS simply being an easy fix for anxiety and risk experienced by frail older people living alone.

### Conclusions

This review reveals how rather simple and well-established telecare technologies such as the PERS are actually complex, integrated caring practices that interact with the different actors involved and create changes in daily living.

The PERS has proven to be durable over time, while many telecare technologies tend never to leave the pilot stage. The reasons for this are complicated, but the results describe some contributing factors. Many users find the PERS to be easy to use and it makes it possible for the end users to live independently by providing help and safety when needed, giving the end users an active role in the caring practice. The PERS in many ways delivers its promises of safety and independent living.

While the Results section describes how the PERS contributes to safety and independence and discusses what the PERS means for the actors involved, it also reveals unforeseen consequences of the alarm and possible improvements in both the device and the service. This review provides us with an understanding of the complexity of practice by showing how even rather simple technology interacts with actors and redefines how they live and work, and even how the technology affects their identities. The Discussion section problematizes this by “zooming out” and argues for an approach to telecare in which the complexity of practice is accounted for, where actors’ resources, attitudes, and abilities are considered when choosing technology.

This paper shows how technology, involved actors, network, and context must be thought of together as part of practice. This calls for a sensitivity to what it means for involved actors when we redistribute responsibility to the end users, and change the roles and work practices of the caring personnel. Another key factor is taking into account how implementing telecare changes the idea of home and all it represents for the actors.

There is a need to be sensitive to diversity in apparently homogenous groups when adopting new telecare technologies in home care practices, and to acknowledge that technology is never neutral. This review shows how understanding end users’ experiences is an important resource for understanding how technology innovations in caring practices are actants in creating new caring practices, thus acknowledging that there are many reasons for resisting and failing to use the technology.

It is time to move away from thinking of telecare technologies as black boxes that can be implemented without changing the caring practice. This review shows how the plug-and-play expectations producers tend to generate is a simplification of the reality. It seems clear that “one size doesn’t fit all.” This calls for a recognition that place and actors matter, and a sensitivity for the practices in which the technology is adopted is necessary.

## Multimedia Appendix 1

Thorough description of the research strategy process.

## Multimedia Appendix 2

Methodological considerations.

## Abbreviations

ED: emergency department
GP: general practitioner
GPS: global positioning system
NTNU: Norwegian University of Science and Technology
PERS: personal emergency response system
RCT: randomized controlled trial
RHQ: reach help quickly
STS: science and technology studies
