# Supplementary material for: The Personal Emergency Response System as a Technology Innovation in Primary Health Care Services: An Integrative Review
Source: J Med Internet Res. 2016 Jul 14;18(7):e187. doi: 10.2196/jmir.5727 (PMC4965612; doi:10.2196/jmir.5727)
Supplement: Supplementary file 1 [file jmir_v18i7e187_app1.pdf]

Multimedia Appendix 1

The following steps describe the research strategy process.

i. Ideally all relevant research should be included to avoid publication bias (Whittemore & Knafl, 2005). There are many different terms describing the PERS. The initial terms detected through pre-search, and expert advice were “Personal Emergency Response System” (PERS), “social alarm”, “safety alarm”, “security alarm,” and “community alarm system”. There was no need to narrow the searches with search strings/ Boolean/Phrase.
The terms “safety telephone”, “safety telephone services”, “medical alert pendant”, “life call/life alert pendant”, “home alarm system”, “telesurveillance”, “personal trigger”, “personal security alarm”, “personal alarm”, “personal response system”, “community alarm service” and “dispersed alarm” were found used in articles from the systematic search, and Google Scholar was also searched for relevance. The 100 most relevant results for each terms, were explored. Broader terms like welfare technology, assistive technology and telecare were not searched since they cover different kinds of technology. Searching ended 1 May 2015.
ii. Searches were conducted in the Cochrane Library, Academic Search Elite, Ageline, Cinahl, ERIC, PubMed, Science Direct, Proquest - all databases, Medline, Embase, Ovid Nursing Database and Clinical Evidence. The databases were selected in collaboration with a university librarian at the university.
iii. For databases using the medical subject heading (Mesh), a variety of terms were used by combining terms indicating the technology device with terms indicating the user of technology in all combinations (search string available on request).
iv. The search engines Google Scholar, NORA and discovery tool Oria and BISYS ASK were used, and the “snowball” methods citation and reference search proved to be important.

v. All articles were reviewed according to inclusion and exclusion criteria.

vi. A data extraction sheet (available on request) inspired by previous studies (Greenhalgh, Robert, Macfarlane, Bate, & Kyriakidou, 2004; Malterud, 2001; Rashman, Withers, & Hartley, 2009) was developed, pilot tested and used. The data extraction sheet included details and evaluation of keywords, aims, themes, methods, analysis, findings, discussions, and references. This was a useful tool for quality assessing the articles. Since the review includes studies with diverse methodology, a methodological quality evaluation as a viable reason for explaining findings was used (Whittemore & Knafl, 2005).
