# Supplementary material for: The Personal Emergency Response System as a Technology Innovation in Primary Health Care Services: An Integrative Review
Source: J Med Internet Res. 2016 Jul 14;18(7):e187. doi: 10.2196/jmir.5727 (PMC4965612; doi:10.2196/jmir.5727)
Supplement: Supplementary file 2 [file jmir_v18i7e187_app2.pdf]

Multimedia Appendix 2

Methodological considerations

Several limitations should be noted. The aim of including all relevant studies is unrealistic due to the number of terms describing the technology.

Mixed methods integrative reviews are complicated to analyse, but the reward is the possibility for a comprehensive result [26]. The ability to draw conclusions from the included studies is further complicated by a diversity in contexts, society, and time. Nevertheless, the integrative review analysis facilitated the summary of findings.

Research methodology in the field has developed over time, corresponding with developments in social science. As a field matures, it becomes more complex, and research questions and methodology change, adding to the scope. Some older included studies were written at a time when requirements for methodology were different. As we can see in this review, the scientific base gives methodological implications directly affecting research focus, and thereby findings.
